# WNT10A Plays an Oncogenic Role in Renal Cell Carcinoma by Activating WNT/β-catenin Pathway

**DOI:** 10.1371/journal.pone.0047649

**Published:** 2012-10-19

**Authors:** Ren-Jun Hsu, Jar-Yi Ho, Tai-Lung Cha, Dah-Shyong Yu, Chieh-Lin Wu, Wei-Ping Huang, Pauling Chu, Ying-Hsin Chen, Jiann-Torng Chen, Cheng-Ping Yu

**Affiliations:** 1 Biobank Management Center of Tri-Service General Hospital, National Defense Medical Center, Taipei, Taiwan; 2 Graduate Institute of Pathology and Parasitology, Tri-Service General Hospital, National Defense Medical Center, Taipei, Taiwan; 3 Graduate Institutes of Life Sciences, National Defense Medical Center, Taipei, Taiwan; 4 Divisions of Urology, Tri-Service General Hospital, National Defense Medical Center, Taipei, Taiwan; 5 Division of Nephrology, Department of Internal Medicine, Tri-Service General Hospital, National Defense Medical Center, Taipei, Taiwan; 6 Department of Emergency Medicine, Tri-Service General Hospital, National Defense Medical Center, Taipei, Taiwan; 7 Department of Ophthalmology, Tri-Service General Hospital, National Defense Medical Center, Taipei, Taiwan; Northwestern University Feinberg School of Medicine, United States of America

## Abstract

Renal cell carcinoma (RCC) is a malignancy with poor prognosis. WNT/β-catenin signaling dysregulation, especially β-catenin overactivation and WNT antagonist silencing, is associated with RCC carcinogenesis and progression. However, the role of WNT ligands in RCC has not yet been determined. We screened 19 WNT ligands from normal kidney and RCC cell lines and tissues and found that WNT10A was significantly increased in RCC cell lines and tissues as compared to that in normal controls. The clinical significance of increase in WNT10A was evaluated by performing an immunohistochemical association study in a 19-year follow-up cohort comprising 284 RCC and 267 benign renal disease (BRD) patients. The results of this study showed that WNT10A was dramatically upregulated in RCC tissues as compared to that in BRD tissues. This result suggests that WNT10A, nuclear β-catenin, and nuclear cyclin D1 act as independent risk factors for RCC carcinogenesis and progression, with accumulative risk effects. Molecular validation of cell line models with gain- or loss-of-function designs showed that forced WNT10A expression induced RCC cell proliferation and aggressiveness, including higher chemoresistance, cell migration, invasiveness, and cell transformation, due to the activation of β-catenin-dependent signaling. Conversely, WNT10A siRNA knockdown decreased cell proliferation and aggressiveness of RCC cells. In conclusion, we showed that WNT10A acts as an autocrine oncogene both in RCC carcinogenesis and progression by activating WNT/β-catenin signaling.

## Introduction

The worldwide incidence of renal cell carcinoma (RCC) is estimated to increase at an annual rate of approximately 2%; moreover, RCC accounts for approximately 1–3% of all adult malignancies. Among patients with RCC, >30% have metastatic RCC; however, only <20% patients show a 5-year survival rate after surgical treatment. In 2008, the incidence rate of RCC was 4/100,000, and its mortality rate was 1.6/100,000 worldwide. In Taiwan, the incidence rate of RCC was 3.2/100,000, and its mortality rate was 1.7/100,000 [1], [2].

*WNT* family genes play important roles in human organogenesis and tumorigenesis; moreover, they are involved in renal development and initiation of several renal diseases [3]–[5]. Nineteen members of *WNT* gene family, which encode secretory cysteine-rich ligands, have been identified in human or mice genomes. These genes can be categorized into 2 classes based on the degree of transformation of mouse mammary epithelial cell line C57MG. The *WNT1* series of genes have a higher ability to transform C57MG and include the *WNT3*, *WNT3A*, and *WNT7A* genes. The other category of genes, i.e., the *WNT5A* series, have moderate or no ability to transform C57MG and include the *WNT2*, *WNT4*, *WNT5A*, *WNT5B*, *WNT6*, *WNT7B*, and *WNT11* genes [6]–[8]. WNT ligands activate 2 intracellular WNT signaling pathways based on β-catenin involvement. In the β-catenin-dependent pathway or canonical pathway, WNT ligands bind to Frizzled receptors resulting in Dishevelled activation. Activated Dishevelled inhibits β-catenin phosphorylation via glycogen synthase kinase-3β adenomatous polyposis coli-axin complex that subsequently inhibits β-catenin degradation, resulting in intracellular β-catenin accumulation and nuclear translocation. Nuclear β-catenin functions as a transcriptional coactivator that complexes with TCF/LEF transcription factors and activates the expression of downstream genes such as cyclin D1, *c-myc*, *TCF-1*, *PPAR-δ*, *MMP-7*, *MMP-26*, and *Axin-2*. Activation of these genes results in increased cell proliferation and differentiation, reduced cell-cell adhesion, enhanced cell migration, and promotion of tumor formation [9]-[11]. In the β-catenin-independent pathway or non-canonical pathway, WNT ligands bind to Frizzled receptors and activate a calcium-dependent pathway that alters cell-cell binding and cell-extracellular matrix binding and prompts the expression of some epithelial-mesenchymal-transition genes. Alternatively, the Rho/JNK pathway is activated, which induces cytoskeleton remodeling and increases cell mobility 12,13.

Aberrant *WNT* gene expression is associated with several types of tumorigenesis. For example, reduction in *WNT1* expression induces apoptosis of many human cancer cells, including non-small cell lung cancer, breast cancer, mesothelioma, sarcoma, and colorectal cancer cells [14], [15]. WNT1 and WNT10B transgenic mice show obvious mammary gland hyperplasia [16], [17]. Furthermore, promoter methylation or other epigenetic modification of *WNT* antagonistic genes such as extracellular antagoinsts (*sFRPs*, *DKKs*, and *WIF1* genes) and cytosolic antagoinsts (*DACTs*, *AXIN2*, and *APC* genes) are also involved in the development of several cancers [18]–[21].

β-Catenin overexpression in RCC is associated with high incidence rate and poor prognosis [22]–[25]. Recently, the association between WNT signaling and RCC had been focused on genetic and epigenetic changes in *WNT* antagonistic genes to determine the association between WNT signaling and RCC [26]. For instance, *DKK2* rs17037102 and *DKK3* rs1472189 polymorphisms are associated with RCC prognosis [27]. Epigenetic silencing of *WNT* antagonistic genes such as *sFRPs* [28]–[33], *DKKs* [34], [35], and *WIF1* [36] is also correlated with poor prognosis in RCC patients. However, WNT ligands participating in RCC carcinogenesis and progression and their mechanism of action have not yet been determined. In this study, we carried out a WNT ligandsscreening in RCC cell lines and tissues. Subsequently the clinical association was evaluated by immunohistochemistry and molecular validation was investigated in cell line models Our results showed that WNT10A played a distinct oncogenic role by activating β-catenin-dependent pathway, which may promote RCC carcinogenesis and progression.

## Materials and Methods

### Ethics Statement

All subjects signed a written informed consent form. All study procedures were approved by the Institutional Review Board of the Tri-Service General Hospital, National Defense Medical Center (TSGH-IRB-098-05-221 and TSGH-IRB-099-05-165).

**Figure 1:**
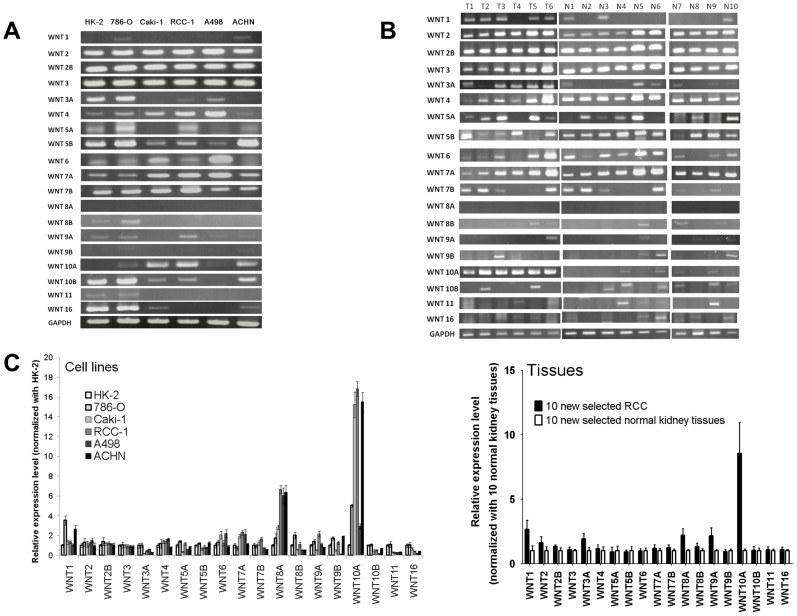
Expression of 19 *WNT* genes in kidney cell lines and tissues. (A) mRNA expression profiles of *WNT* genes from kidney cell lines obtained using RT-PCR. mRNA expression profiles of 19 *WNT* genes from 5 RCC cell lines (786-O, Caki-1, RCC-1, A498, and ACHN) and 1 immortalized proximal tubule epithelial cell line from a normal adult human kidney (HK-2) were obtained using RT-PCR. Higher WNT10A expression was observed in RCC cell lines Caki-1, RCC-1, and ACHN, but lower expression was observed in RCC cell lines 786-O and A498. WNT10A expression was undetectable in the normal kidney cell line HK-2. (B) mRNA expression profile of *WNT* genes from BRD and RCC specimens obtained using RT-PCR. mRNA expression profiles of 19 *WNT* genes from 6 paired RCC (T1–T6) and paratumoral (N1–N6) tissues. mRNA expression profiles of *WNT* genes from other 4 BRD tissues (N7–N10) were also examined. Higher expression of WNT10A was observed in most RCC tissues than in paratumoral and BRD tissues. (C) Quantitative real-time PCR for detecting the expression of *WNT* genes. Expression of *WNT* genes in each cell line was determined using SYBR Green real-time PCR. An independent data set comprising 10 RCC and 10 normal kidney tissues was used. The results obtained were similar to those obtained for RT-PCR, i.e., higher expression of WNT10A was observed in most RCC cell lines and tissues than in normal controls. All experiments were performed in triplicate. Relative expression of each gene from each cell line was normalized with that of the gene from HK-2 normal kidney cell line; relative expression of each gene of tissues was normalized with the mean of 10 normal kidney tissues.

### Subjects

The study cohort comprised 284 RCC [37] and 267 benign renal disease (BRD) patients selected from an initial population of 3,494 patients who were treated at the Tri-Service General Hospital from 1988–2006 and in whom the diseases were diagnosed histologically by examining specimens obtained by excisional biopsy or therapeutic surgical resection (summarized in Figure S1). Clinical and histological information of patients was obtained from their charts and pathological reports, which are summarized in Tables 1, 2, and S3. Tumor staging was performed according to the American Joint Committee on Cancer tumor-node-metastasis staging system, and histological grading was performed according to the World Health Organization classification criteria. All formalin-fixed, paraffin-embedded specimens were resected, stained with H&E, and re-examined by CPY who was blinded to the original diagnosis and subsequent outcome. Representative specimens were spotted for tissue microarray (TMA) construction. All specimens and clinical information of patients were obtained from the Tissuebank of Tri-Service General Hospital, National Defense Medical Center.

**Figure 2:**
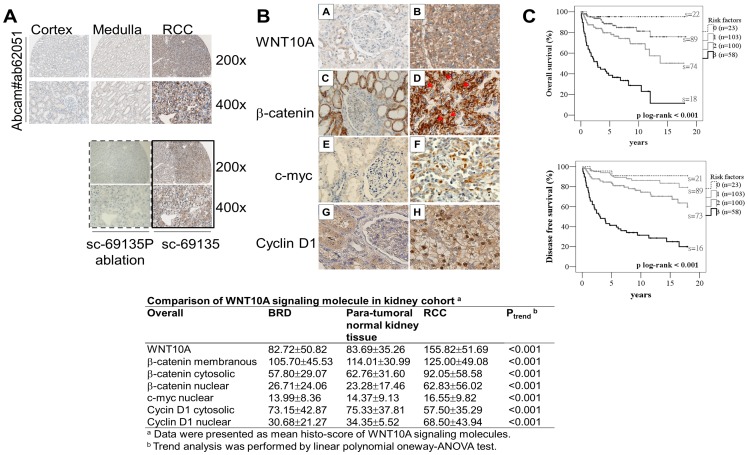
Immunohistochemical staining of WNT10A, β-catenin, and cyclin D1 in kidney tissues. (A) WNT10A showed very low cytoplasmic expression in renal tubular cells of non-tumoral tissues from both the cortex and medulla. Moreover, WNT10A showed a dramatically higher cytoplasmic expression in RCC tissues. To confirm WNT10A immunohistochemical profiles, WNT10A antibody (sc69135) was used for a serial tissue microarray section, and antibody ablation was performed using its competitive peptide (sc69135P). WNT10A antibody (sc69135) showed similar profiles, and antibody ablation with WNT10A peptide abolished the staining pattern (sc69135P). (B) WNT10A showed higher cytoplasmic expression in RCC cells (A: cortex, B: CCRCC; 400×). β-catenin showed higher intracellular accumulation (cytoplasmic and nuclear; red arrows indicate some representative nuclear-stained cells) in RCC tissues (C: cortex, D: CCRCC; 400×). However, β-catenin showed higher membranous expression in renal tubular cells from non-tumoral normal kidney tissue. Moreover, c-myc (E: cortex, F: CCRCC; 400×) and cyclin D1 (G: cortex, H: CCRCC; 400×) showed higher nuclear expression in RCC tissues, but cyclin D1 showed weak cytoplasmic expression in renal tubular cells from non-tumoral normal kidney tissue. (C) Kaplan-Meier analysis of WNT10A, nuclear β-catenin, and nuclear cyclin D1 accumulative effects on overall survival (OS) and disease-free survival (DFS). There exists an accumulated dose effect, in that patients with higher WNT10A, nuclear β-catenin, and nuclear cyclin D1 levels have poor RCC prognosis (n  =  initial patient number, s  =  number of survivors at the end of the study). The 4 groups were defined as follows: 0, carriers with lower expression of all the 3 markers; 1, carriers with higher expression of any 1 of the 3 markers; 2, carriers with higher expression of any 2 of the 3 markers; 3, carriers with higher expression of all the 3 markers. *P* of log-rank test <0.001.

### Reverse Transcriptase Polymerase Chain Reaction (RT-PCR) and Quantitative Real-time PCR of *WNT* Family Genes

Total RNA of kidney or RCC cells and tissues was isolated using TRIzol (Invitrogen). RNA samples were treated with RQ1 RNase-free DNase (Promega), according to the manufacturer’s instructions, to remove any genomic DNA contamination. Five micrograms of treated RNA samples were reverse transcribed using SuperScript III (Invitrogen). RT-PCR was performed using 20 µL of reaction mixture containing 2 µL of cDNA, 5 pmol of each primer, 2 U of recombinant Taq DNA polymerase (Invitrogen), 1× reaction buffer, and 200 pmol of dNTPs. Amplification was performed for 35 cycles under the following conditions: denaturation at 94°C for 1 min, annealing at 55°C for 30 sec, extension at 72°C for 1 min, and final elongation at 72°C for 7 min. PCR products were separated by electrophoresis on a 2% agarose gel and visualized by staining with ethidium bromide. SYBR Green quantitative real-time PCR was performed using StepOne Real-Time PCR System (Applied Biosystems) with Maxima Hot Start PCR Master Mix (2×) (Fermantus). Glyceraldehyde-3-phosphate dehydrogenase was used as an internal control. In addition to the analysis of melting curve, real-time PCR products were analyzed by gel electrophoresis to confirm single PCR products. Primer sets uesd in RT-PCR and real-time PCR are listed in Table S1.

**Table 1:** Association of RCC risk factor and WNT signaling molecules.

Subject characteristics	RCC	BRD	Univariate		Multivariate	
	(n = 284)	(n = 267)	RR (95%CI)	*p* †	RR (95% CI)	*p* †
**WNT signaling markers**						
WNT10A *						
Cytosolic - low	96	149	(reference)		(reference)	-
- high	188	118	**2.473 (1.752–3.490)**	**<0.001**	**2.267 (1.466–3.504)**	**0.001**
β-catenin ^a^						
Membraneous - low	113	129	(reference)		(reference)	
- high	171	138	**1.415 (1.009–1.983)**	**0.044**	1.360 (0.856**–**2.161)	0.192
Cytosolic - low	145	180	(reference)		(reference)	
- high	139	87	**2.163 (1.526–3.066)**	**<0.001**	1.329 (0.847**–**2.085)	0.215
Nuclear - low	155	218	(reference)		(reference)	
- high	129	49	**3.703 (2.512–5.458)**	**<0.001**	**2.820 (1.765–4.505)**	**<0.001**
c-myc ^a^						
Nuclear - low	181	193	(reference)		(reference)	
- high	103	74	**1.484(1.034–2.129)**	**0.032**	0.966 (0.612**–**1.527)	0.883
Cyclin D1^a^						
Cytosolic - low	161	142	(reference)			
- high	123	125	0.868 (0.620**–**1.214)	0.408	n.a.	n.a.
Nuclear - low	124	210	(reference)		(reference)	
- high	160	57	**4.754 (3.267–6.917)**	**<0.001**	**3.919 (2.599–5.908)**	**<0.001**
**Demographic factors**						
Age	59.44±13.79	53.86±18.52	**1.021 (1.011–1.032)**	**<0.001**	1.013 (0.998–1.027)	0.062
Gender – Female	98	141	(reference)			
Male	186	126	**2.124 (1.507**–**2.993)**	**<0.001**	**2.972 (1.972**–**4.479)**	**<0.001**

* IHC results were categorized as low (≤mean) and high (>mean) based on the values in Figure 2B.

† Based on the logistic regression model, with statistical significance (*p*<0.05) was shown in boldfaced. n.a.: not anaylzed.

**Table 2:** Univariate and multivariate analyses of prognostic factors and RCC survival.

		Overall survival (OS)				Disease-free survival (DFS)			
Subject characteristics		Univariate		Multivariate		Univariate		Multivariate	
	n	RR (95%CI)	*p* †	RR (95%CI)	*p* †	RR (95%CI)	*p* †	RR (95% CI)	*p* †
**WNT signaling markers**									
WNT10A *									
Cytosolic low	96	(reference)		(reference)		(reference)		(reference)	
high	188	**4.762 (2.450–9.255)**	**<0.001**	**2.537 (1.183–5.441)**	**0.017**	**4.379 (2.322–8.259)**	**<0.001**	**2.733 (1.284–5.820)**	**0.009**
β-catenin *									
Membraneous low	113	(reference)		(reference)		(reference)		(reference)	
high	171	0.704 (0.455**–**1.089)	0.115	n.a.	n.a.	0.734 (0.479**–**1.124)	0.155	n.a.	n.a.
Cytosolic low	145	(reference)		(reference)		(reference)		(reference)	
high	139	**2.180 (1.367–3.478)**	**0.001**	1.004 (0.469**–**2.150)	0.992	**2.232 (1.419–3.510)**	**0.001**	1.046 (0.495**–**2.212)	0.906
Nuclear low	155	(reference)		(reference)		(reference)		(reference)	
high	129	**4.160 (2.509–6.896)**	**<0.001**	**2.281 (1.083–4.804)**	**0.030**	**4.115 (2.531–6.689)**	**<0.001**	**2.397 (1.166–4.924)**	**0.017**
c-myc *									
Nuclear low	181	(reference)		(reference)		(reference)		(reference)	
high	103	**1.575 (1.019–2.436)**	**0.041**	0.757 (0.395**–**1.449)	0.401	**1.660 (1.085–2.541)**	**0.020**	0.860 (0.454**–**1.627)	0.642
cyclin D1 *									
Cytosolic low	161	(reference)		(reference)		(reference)		(reference)	
high	123	0.668 (0.430**–**1.075)	0.098	n.a.	n.a.	0.693 (0.444**–**1.081)	0.106	n.a.	n.a.
Nuclear low	124	(reference)		(reference)		(reference)		(reference)	
high	160	**1.660 (1.045–2.635)**	**0.032**	**2.110 (1.128–3.947)**	**0.019**	**1.708 (1.087–2.686)**	**0.020**	**2.446 (1.322–4.524)**	**0.004**
**Demographic factors**									
Age		0.995 (0.979**–**1.011)	0.546	n.a.	n.a.	0.995 (0.980**–**1.011)	0.559	n.a.	n.a.
Gender – Male	186	1.340 (0.832–2.160)	0.229	n.a.	n.a.	1.451 (0.904–2.327)	0.123	n.a.	n.a.
Female	98								
Lateriality left	158	1.132 (0.732–1.752)	0.578	n.a.	n.a.	1.106 (0.722–1.694)	0.645	n.a.	n.a.
Right	126								
Histopathology –CCRCC	230	(reference)				(reference)			
PRCC	34	1.579 (0.846–2.947)	0.152	n.a.	n.a.	1.547 (0.832–2.879)	0.170	n.a.	n.a.
ChRCC	20	0.988 (0.425–2.298)	0.977	n.a.	n.a.	0.982 (0.424–2.274)	0.961	n.a.	n.a.
TNM stage I+II	163	(reference)				(reference)			
III+IV	121	**5.627 (3.123**–**10.140)**	**<0.001**	**2.492 (1.290**–**4.814)**	**0.007**	**5.800 (3.271**–**10.283)**	**<0.001**	**2.510 (1.336**–**4.718)**	**0.004**
Grade G1+G2	117	(reference)				(reference)			
G3+G4	167	**3.935 (2.162**–**7.161)**	**<0.001**	**2.560 (1.271**–**5.157)**	**0.009**	**3.568 (2.033**–**6.262)**	**<0.001**	**2.544 (1.296**–**4.992)**	**0.007**
Chemotherapy No	258	(reference)				(reference)			
Yes	26	1.589 (0.841–3.004)	0.154	n.a.	n.a.	1.598 (0.848–3.012)	0.147	n.a.	n.a.
Radiotherapy No	232	(reference)				(reference)			
Yes	52	1.409 (0.847–2.344)	0.187	n.a.	n.a.	1.537 (0.937–2.523)	0.089	n.a.	n.a.

* Categorized as low (≤mean) and high (>mean) based on the values in Figure 2B.

† Analyzed with Cox harzard regression model, and the statistic significance (p<0.05) is showed in boldfaced. (n.a.: not analyzed).

### Cell Lines

Human RCC cell line A498 (derived from papillary RCC); ACHN, Caki-1, and 786-O (all derived from clear cell RCC); and immortalized proximal tubule epithelial cell line (HK-2) derived from normal adult human kidney were obtained from Bioresource Collection and Research Center (BCRC, Taiwan). RCC-1 cell line (derived from clear cell RCC) was obtained from Dr. DS Yu (Division of Urology, Tri-Service General Hospital); this cell line was derived from primary culture. Collection and maintenance of primary tissue/cell culture were approved by the Institutional Review Board of the Tri-Service General Hospital, National Defense Medical Center (TSGH-IRB-098-05-221) under the project “Establishment and Maintenance of the Tissue Bank of Tri-Service General Hospital.” RCC-1 and A498 were maintained in Dulbecco’s Modified Eagle medium (DMEM) containing 10% fetal bovine serum (FBS), 1 µg/mL penicillin, and 1 µg/mL streptomycin at 37°C in humidified 5% CO_2_ atmosphere. ACHN, Caki-1, and 786-O were maintained in RPMI-1640 medium containing 10% FBS, and HK-2 was maintained in keratinocyte serum-free medium containing 0.1 ng/mL recombinant epidermal growth factor and 20 µg/mL bovine pituitary extract (cell culture mediums and supplements were all obtained from Invitrogen) under the same antibiotic and culture conditions as that used for RCC-1 and A498.

**Figure 3:**
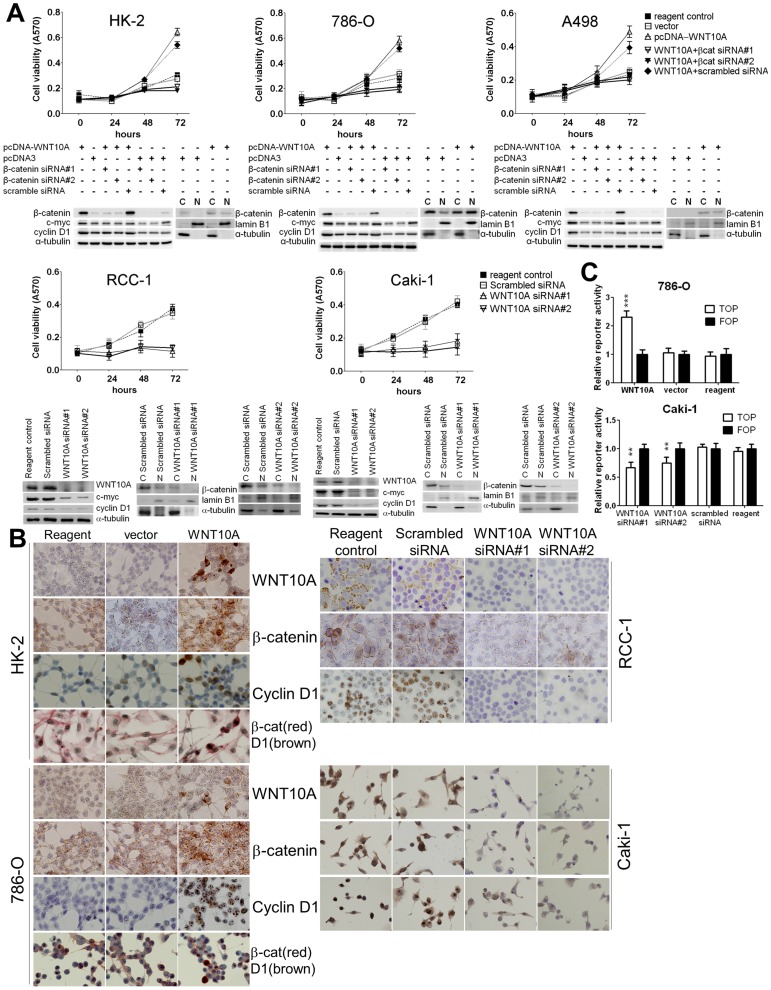
Forced WNT10A expression and WNT10A siRNA knockdown in kidney cell lines. (A) Cell growth curve of pcDNA-WNT10A-transfected cells and WNT10A siRNA knockdown cells. WNT10A gain-of-function was achieved by transfecting pcDNA-WNT10A in normal kidney cell line HK-2 and RCC cell lines 786-O and A498, which had relatively lower endogenous WNT10A expression. Cotransfection of pcDNA-WNT10A and β-catenin siRNA was also performed. Conversely, WNT10A loss-of-function was achieved by WNT10A siRNA knockdown in RCC-1 and Caki-1, which have higher endogenous WNT10A. After transient transfection for 48 h, cell viability was determined using the MTT assay and was compared with that of cells transfected with Lipofectamine only (reagent control), pcDNA3.1 vector only (vector), and pcDNA-WNT10A and with that of cells cotransfected with pcDNA-WNT10A and β-catenin siRNA at 12, 24, 48, and 72 h. HK-2, 786-O, and A498 transfected with pcDNA-WNT10A showed significant increase in cell proliferation after 48 h; however, cotransfection of pcDNA-WNT10A and β-catenin siRNA reduced WNT10A-induced cell proliferation. Conversely, RCC-1 and Caki-1 transfected with WNT10A siRNA showed significant decrease in cell proliferation as compared to cells transfected with reagent and scrambled siRNA controls. (**P<0.001, *P<0.05; Student’s *t*-test). Western blot analysis of pcDNA-WNT10A- and WNT10A siRNA-transfected cells from each cell line was also performed. HK-2, 786-O, and A498 were transfected with 3 µg of pcDNA-WNT10A, pcDNA3.1 vector alone, and cotransfected with β-catenin siRNA for 48 h. Conversely, RCC-1 and Caki-1 were transfected with 1 µg WNT10A siRNA or scrambled siRNA controls for 72 h. Twenty micrograms of total protein extract from each cell line was loaded onto SDS-polyacrylamide gel and western blot analysis was performed. Forced WNT10A expression in HK-2, 786-O, and A498 remarkably increased the concentration of WNT10A than that of the vector control in these cells. Forced WNT10A expression also upregulated nuclear β-catenin, cyclin D1, and c-myc levels, and cotransfection with β-catenin siRNA reduced cyclin D1 and c-myc expression levels. Conversely, WNT10A siRNA knockdown in RCC-1 and Caki-1 markedly reduced endogenous WNT10A levels, thus reducing nuclear β-catenin, cyclin D1, and c-myc levels. (B) Immunocytochemical analysis of pcDNA-WNT10A-transfected cells and WNT10A siRNA knockdown cells. After pcDNA-WNT10A was transfected in HK-2 and 786-O, expression of WNT10A, β-catenin, and cyclin D1 was determined by immunocytochemistry. WNT10A levels were significantly increased in the transfected cells. β-catenin showed high intracellular accumulation in WNT10A-transfected cells and low membranous expression in vector- and reagent-transfected controls. Moreover, cyclin D1 was upregulated in the nucleus of pcDNA-WNT10A-transfected cells but showed low cytoplasmic expression in vector- and reagent-transfected controls. Upregulated nuclear β-catenin and cyclin D1 were also observed in the same pcDNA-WNT10A-transfected cells (red staining, β-catenin; brown staining, cyclin D1). Conversely, WNT10A siRNA-transfected RCC-1 and Caki-1 showed an obvious reduction in endogenous WNT10A expression and reduced intracellular β-catenin accumulation and cyclin D1 expression. (C) TCF/LEF reporter assay. Forced WNT10A expression in 786-O for 48 h significantly induced luciferase activity in these cells than that in vector- and reagent-transfected controls. Conversely, WNT10A siRNA-transfected Caki-1 showed significantly reduced luciferase activity after 72 h as compared to that in vector- and reagent-transfected controls.

### Transfection of pcDNA3-WNT10A Plasmids or siRNAs Containing WNT10A and β-catenin

Cell lines were seeded in a 6-cm dish at density of 5×10^5^ cells/dish and incubated overnight. HK-2, 786-O, and A498 were prepared for gain-of-function design, and RCC-1 and Caki-1 were prepared for loss-of-function design. Three micrograms of pcDNA3-WNT10A plasmid (constructed by laboratory processing) or 1 µg each of WNT10A siRNA (s37228 and s37230; Invitrogen) and β-catenin siRNA (sc29209 and sc44252; Santa Cruz) were added to Opti-MEM with Lipofectamine 2000 (Invitrogen) for transfection, according to the manufacturer’s instructions. After 12 h of incubation, the old medium was replaced with fresh DMEM containing 10% FBS. Cells were harvested at 48 h after transfection for the pcDNA-WNT10A group and at 72 h after transfection for the WNT10A and β-catenin siRNA groups. Harvested cells were subjected to RT-PCR, real-time PCR, western blot analysis, and immunocytochemistry.

**Figure 4:**
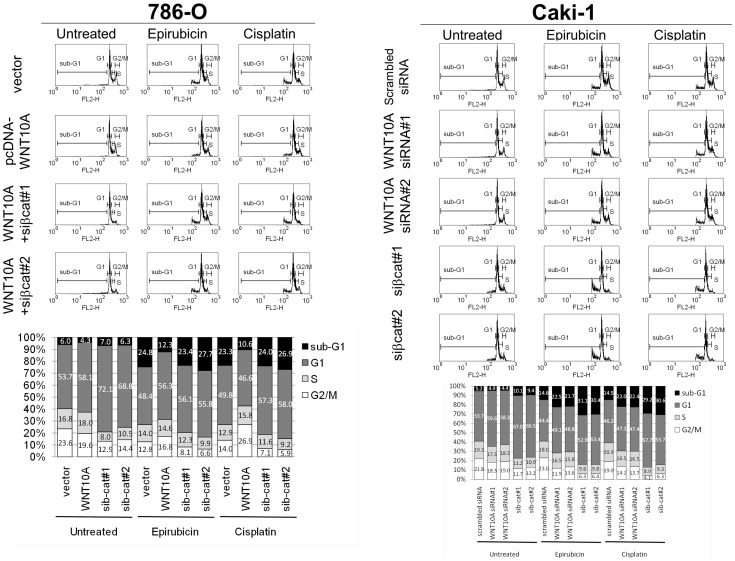
Forced WNT10A expression induced higher chemoresistance in 786-O, and WNT10A siRNA knockdown reduced chemoresistance in Caki-1. Effects of WNT10A on cell survival were analyzed using PI staining with flow cytometry. pcDNA-WNT10A-transfected 786-O showed slight increment in G1 phase as compared to that observed in vector-transfected controls after 48 h of solvent (DMSO) treatment. Moreover, cotransfection of pcDNA-WNT10A and β-catenin siRNA showed an obvious G1 arrest (left lane of 786-O). However, pcDNA-WNT10A-transfected 786-O showed lower sub-G1 cell population than vector-transfected controls after 48 h of treatment with 2 µM epirubicin (middle lane of 786-O) or 10 µM cisplatin (right lane of 786-O). Besides, cotransfection of pcDNA-WNT10A and β-catenin siRNA increased the chemosensitivity of 786-O. Conversely, WNT10A siRNA-transfected Caki-1 showed minor sub-G1 and G1 phase modifications as compared to those observed in scrambled siRNA-transfected controls. However, β-catenin siRNA induced both higher sub-G1 and G1 arrest (left lane of Caki-1). WNT10A siRNA- and β-catenin siRNA-transfected Caki-1 showed significantly increase sub-G1 cell population than scrambled siRNA-transfected controls after 48 h of treatment with 2 µM epirubicin (middle lane of Caki-1) or 10 µM cisplatin (right lane of Caki-1). The proportion of each cell cycle phase belonging to both cell lines are shown using bar charts.

**Figure 5:**
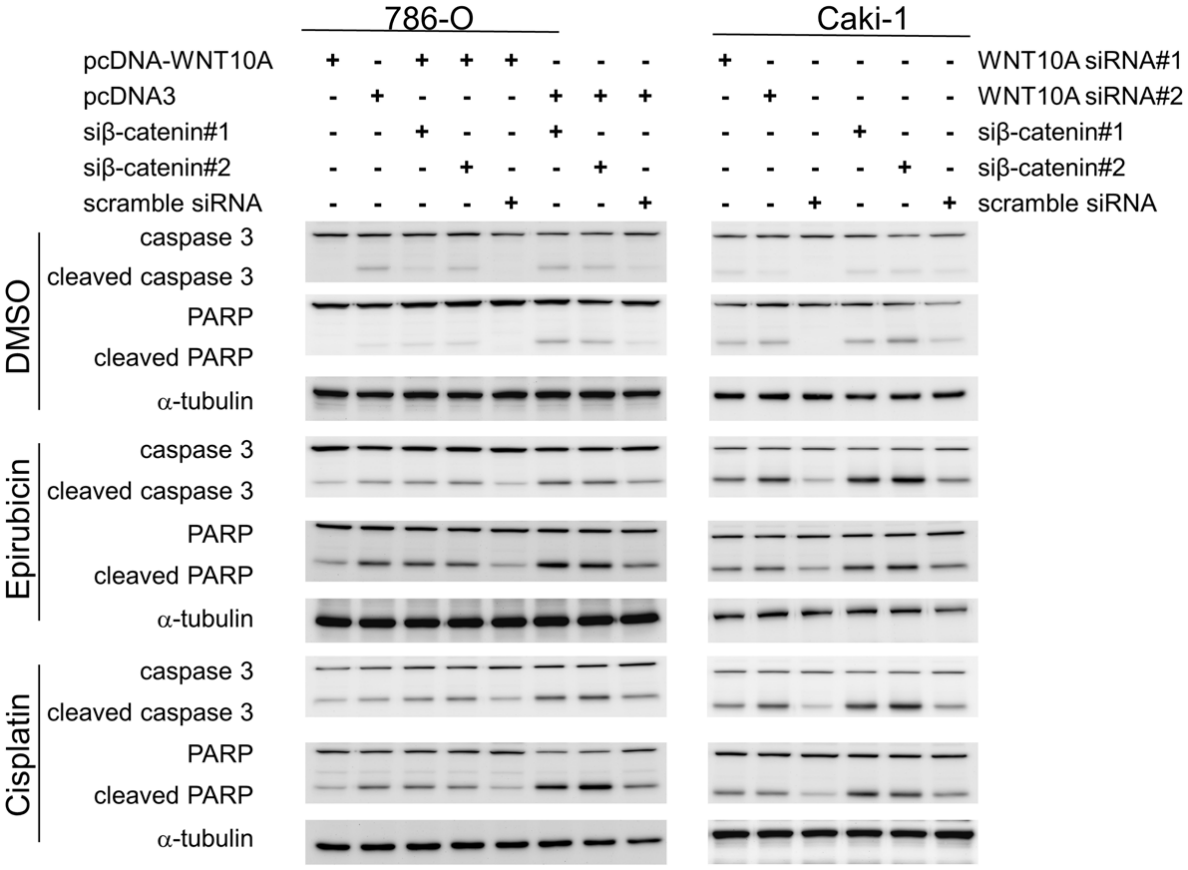
Western blot analysis of apoptotic markers. Effects of WNT10A on cell survival were evaluated by western blot analysis of apoptotic markers. pcDNA-WNT10A-transfected 786-O showed a slightly reduced basal level of cleaved caspase 3 and PARP as compared to that observed in vector-transfected controls after 48 h of solvent (DMSO) treatment. However, β-catenin siRNA-transfected 786-O showed higher levels of cleaved caspase 3 and PARP than those observed in other groups of cells. After 48 h of treatment with 2 µM epirubicin or 10 µM cisplatin, pcDNA-WNT10A-transfected 786-O showed lower levels of cleaved caspase 3 and PARP than those observed in vector-transfected controls or cells cotransfected with pcDNA-WNT10A and β-catenin siRNA. Conversely, both WNT10A siRNA- and β-catenin siRNA-transfected Caki-1 showed higher levels of cleaved caspase 3 and PARP than those observed in scrambled siRNA-transfected controls. β-catenin silencing seemed to induce the production of cleaved caspase 3 and PARP. Both WNT10A siRNA- and β-catenin siRNA-transfected Caki-1 showed significantly higher levels of cleaved caspase 3 and PARP than those observed in scrambled siRNA-transfected controls after 48 h of treatment with 2 µM epirubicin or 10 µM cisplatin.

### Western Blot Analysis

Transfected cells were washed twice with 1×phosphate buffered saline (PBS) and lysed in 100 µL of 1× RIPA lysis buffer (50 mM Tris-HCl, pH 7.4, 150 mM NaCl, 1 mM EDTA, 1% Triton X-100, 1% sodium deoxycholate, and 0.1% sodium dodecyl sulfate (SDS); Millipore) containing 1× protease inhibitor (Roche, #04693116001). Thirty micrograms of protein from the supernatant was loaded onto a SDS polyacrylamide gel, and western blot analysis was performed to detect WNT10A (Abcam, #ab62051), β-catenin (Epitomics, #1247-1), and cyclin D1 (Cell signaling DCS-6, #2926) levels. Immunoreactive bands were developed using ECL system (Millipore) and were quantified using UVP BioSpectrum Imaging System.

### Immunohistochemistry and Immunocytochemistry

Constructed tissue microarray sections containing 100 individual 2-mm diameter samples per array were subjected to immunohistochemical analysis. Four micrometer TMA sections were blocked with 10% goat serum for 1 h and incubated with WNT10A (Abcam, #ab62051), β-catenin (Epitomics, #1247-1), c-myc (Cell signaling, D84C12 #5605), and cyclin D1 (Cell signaling, DCS-6 #2926) antibodies (1∶200 dilution for each) for 2 h at room temperature. Slides were washed 3 times with 1× TBST (10 mM Tris, pH 7.4, 150 mM NaCl, and 0.1% Tween-20) for 10 min, processed using Super Sensitive™ Polymer HRP Detection System/DAB (Biogenex, #QD410-YAXE), and counterstained using hematoxylin. For confirming WNT10A immunohistochemical profiles, WNT10A antibody (sc-69135, 1∶50 dilution) was used on serial TMA sections, and antibody ablation was performed using a competitive peptide (sc-69135P). Results were interpreted by an experienced researcher (JYH) and a senior pathologist (CPY) who were blinded to the experimental and the associated clinical pathological data. Positive cell percentage and intensity were recorded to calculate histoscore (histoscore  =  positive cell percentage × intensity; intensity is divided into 4 ranks: negative, weak, moderate, and strong) [38]. Membranous, cytoplasmic, or nuclear staining scores were separately recorded once for each specimen. For immunocytochemical analysis, approximately 2×10^4^ cells were seeded on an 18×18-mm cover glass. After transfection, cells were resuspended, counted, and reseeded on a cover glass and incubated for 16 h. Next, the cells were washed twice at the indicated time with 1×PBS (200 mM NaCl, 3 mM KCl, 10 mM Na_2_PO_4_, and 1.5 mM KH_2_PO_4_, pH 7.4), fixed in acetone/methanol (1∶1) at −20°C for 30 min, and permeabilized with 0.1% Triton X-100 in 1× PBS at room temperature for 10 min. The cells were then washed 3 times with 1× TBST and blocked in 10% goat serum for 1 h. After incubation with the previously mentioned antibodies (1∶200 dilution for each) for 2 h at room temperature, the cells were washed 3 times with 1× TBST for 10 min, stained using the Super Sensitive Polymer HRP Detection System/DAB (Biogenex), and counterstained with hematoxylin. For dual immunostaining, DAB (brown) was used as the chromogen for cyclin D1 and AEC was used as the chromogen for β-catenin.

**Figure 6:**
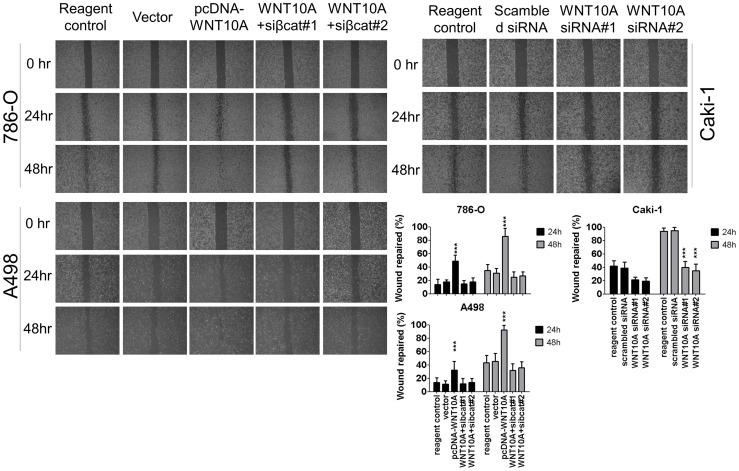
WNT10A promotes wound healing ratio in RCC cells. After 48 h of transfection with 3 µg pcDNA-WNT10A or WNT10A siRNA, cells were scraped with p200 tip (time 0) and photographed each day. Forced WNT10A expression in 786-O and A498 increased the migration ability of these cells in the wound healing assay, especially 48 h after scraping. Moreover, cotransfection of pcDNA-WNT10A and β-catenin siRNA reduced WNT10A promotive effect on cell migration (*P*<0.001, Student’s *t*-test between pcDNA-WNT10A-transfected cells and other cell groups). Conversely, each WNT10A siRNA knockdown in Caki-1 reduced the migration ability of these cells in the wound healing assay (*P*<0.001 at 48 h after scraping, Student’s *t*-test between WNT10A siRNA-transfected cells and scrambled siRNA-transfected controls or between WNT10A siRNA-transfected cells and reagent-transfected controls). Wound repair percentage of each cell line is shown using bar charts.

**Figure 7:**
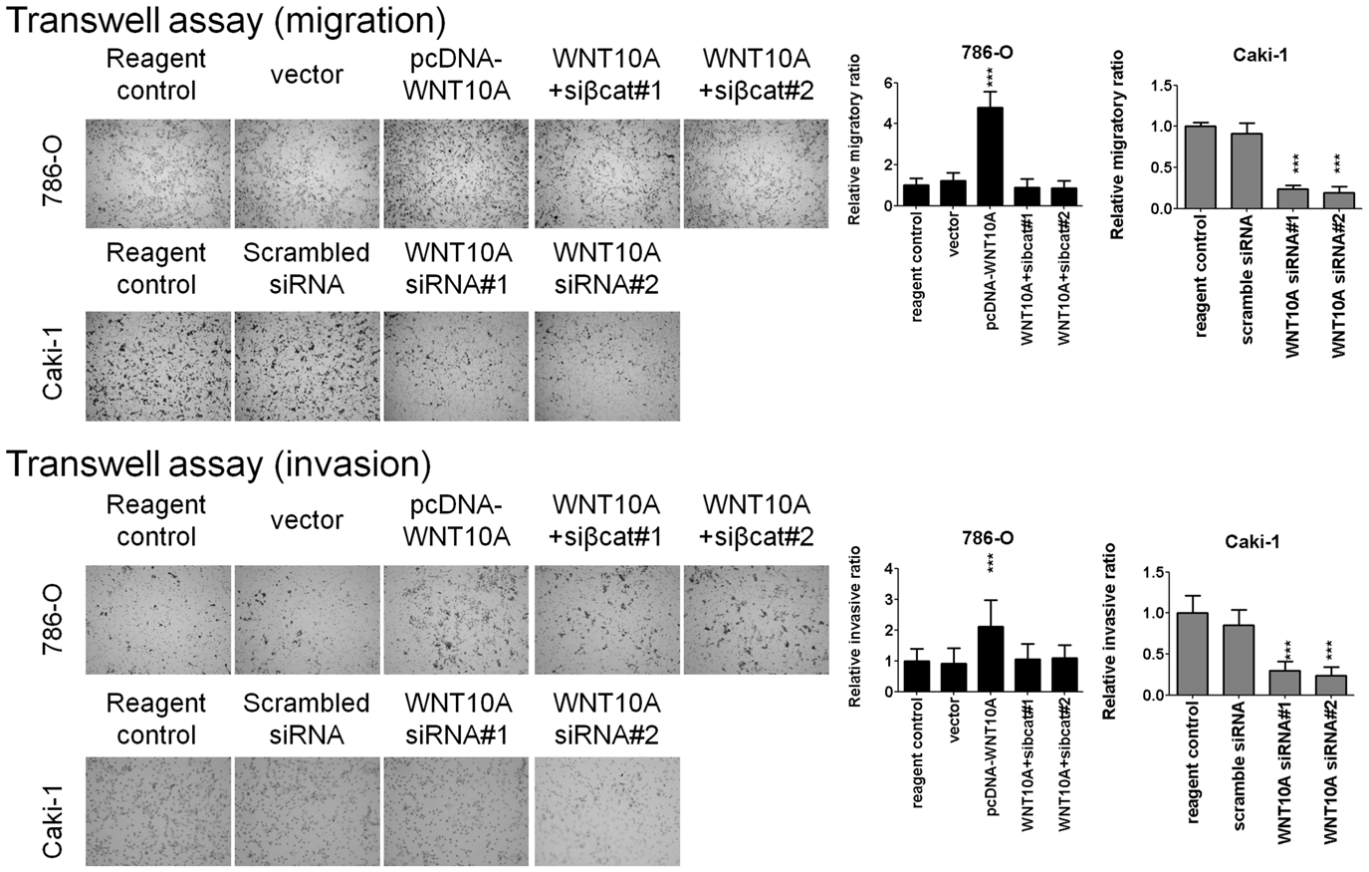
WNT10A promotes RCC cell migration and invasion in transwell assay. Transwell assay was used to evaluate the effects of WNT10A on cell migration. After 48 h of transfection, 1×10^4^ cells were transferred into 8-µm inserts and incubated for another 48 h. Forced WNT10A expression in 786-O significantly increased the migration ability of these cells through the transwell; however, cotransfection with β-catenin siRNA reduced the WNT10A promotive effect on cell migration. Conversely, WNT10A siRNA knockdown in Caki-1 significantly reduced the migration ability of these cells. Bar charts show the cell migration ratio; significances were analyzed using the Student’s *t*-test. Invasion assay took advantage of the Matrigel-coated transwell assay. After 48 h of transfection, 2×10^4^ cells were transferred into 1 mg/mL Matrigel-coated insert and incubated for another 48 h. Forced WNT10A expression in 786-O significantly increased the invasive ability of these cells, but cotransfection with β-catenin siRNA reduced the WNT10A promotive effect on cell invasion. Conversely, WNT10A siRNA knockdown in Caki-1 significantly reduced the invasive ability of these cells. Bar charts show the cell invasion ratio; and significances were analyzed as mentioned above.

**Figure 8:**
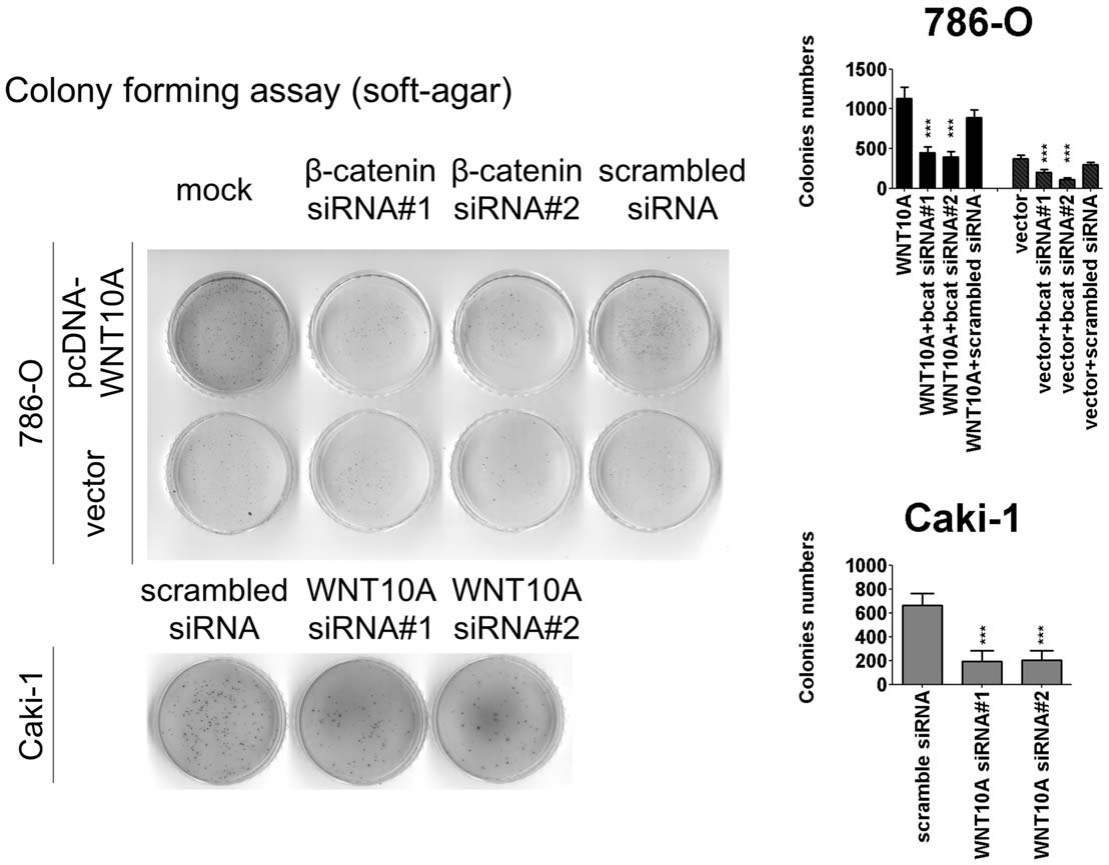
WNT10A increases colony formation ability. Colony formation assay was used to evaluate the effect of WNT10A on cell transformation. After 48 h of transfection, 2×10^4^ cells were mixed with 0.3% agarose in 1× complete RPMI and transferred into a coated 6-cm dish, as described in Materials and Methods. To prolong the effects of transfection, pcDNA-WNT10A-transfected cells were maintained in 200 µg/mL G418, and WNT10A siRNA or β-catenin siRNA knockdown cells were maintained in 10 nM of WNT10A siRNA for 15 days. Forced WNT10A expression in 786-O significantly increased the number of colonies of these cells; cotransfection with β-catenin siRNA reduced the WNT10A promotive effect on colony formation ability. Moreover, each WNT10A siRNA knockdown in Caki-1 reduced the number of colonies of these cells. Bar charts show the number of colonies; significances were analyzed by using the Student’s *t*-test.

### TCF/LEF Reporter Assay

We used TCF/LEF reporter assay with luciferase reporter plasmids (Super 8× TOPFlash, containing wild-type TCF binding sites, and Super 8× FOPflash, containing mutant TCF binding sites; Addgene, #clones M50 and M51) to detect the activity of WNT/β-catenin signal transduction. pGL4.71 Renilla luciferase vector (Promega) was cotransfected to normalize transfection efficiency. Lipofectamine 2000 (Invitrogen) was used for transfection, as described above. Luciferase activity was assayed at 48 h after transfection by using a Dual-Luciferase Reporter Assay System (Promega). All the experiments were performed in triplicate.

### Cell Viability Assay

Cells transfected with pcDNA3-WNT10A plasmid, WNT10A siRNA, β-catenin siRNA, vector control, and scrambled siRNA control were washed twice with PBS at the indicated time and subjected to 3-(4, 5-dimethylthiazol-2-yl)-2, 5-diphenyltetrazolium bromide (MTT) assay for the detection of cell viability. In brief, 100 µL of 2 mg/mL MTT reagent was added to each well and incubated at 37°C for 3 h, and absorbance was read at 570 nm. A_570_ was recorded at 0, 24, 48, and 72 h. For each indicated time and condition, reading was taken in 6 repeats.

### Flow Cytometry

For determining the effects of WNT10A on apoptosis, pcDNA-WNT10A-, pcDNA3.1 vector control-, and β-catenin siRNA-transfected 786-O (after reaching >70% confluence) were treated with 2 µM epirubicin or 10 µM cisplatin in a fresh culture medium. WNT10A siRNA-, β-catenin siRNA-, or scrambled siRNA control-transfected Caki-1 were also administered the same treatment. Since epirubicin is a water-soluble compound, the untreated controls were treated with equal DMSO concentration of cisplatin group. After 48 h, the treated cells were trypsinized, washed with 1× PBS, and resuspended. A total of 1×10^6^ cells were fixed with 100% EtOH for 10 min and then incubated with 1 mg/mL propidium iodide (PI) for 10 min at room temperature. Cells were filtered using 40-µm Cell Strainer (BD Falcon) and analyzed using a BD FACSCalibur (BD Biosciences) within 20 min of staining.

### Cell Migration and Invasion Assay

For wound-healing experiments, cells were plated in 6-well plates and cultured to 90% confluence. Cells were scraped using a p200 tip (time 0), and suspended cells were washed before obtaining each image. Migration distance was measured from images (5 fields) taken at each indicated time point. Transwell assay of Caki-1 and 786-O was assessed using 8-µm inserts (BD Biosciences). A total of 1×10^4^ cells were suspended in 100 µL serum-free RPMI-1640 media and loaded into upper wells; lower chambers were filled with 500 µL of complete medium (RPMI-1640 supplemented with 10% FBS). For invasion assay, each insert was coated with 1 mg/mL Matrigel and incubated at 37°C for 5 h. A total of 2×10^4^ cells were suspended in 100 µL serum-free RPMI-1640 media and loaded into coated inserts; lower chambers were filled with the complete medium. Migration and invasion chambers were incubated in a humidified 5% CO_2_ incubator at 37°C for 48 h. Cells were then fixed with 500 µL of methanol for 15 min. The inner surface of the upper chambers was wiped using cotton swabs to remove non-migrated cells in the migration assay or scrapes of Matrigel in the invasion assay. The chambers were then washed with 500 µL PBS and stained with 500 µL hematoxylin for 1 min at room temperature. After washing again with 500 µL PBS, the transwell membranes were torn and kept in slides. Stained cells were counted using the Image J software, and 5 random fields were counted at 100× magnification.

### Colony Formation Assay

Transfected 786-O and Caki-1 were used in the colony formation assay. 786-O were transfected with pcDNA-WNT10A, vector control, or cotransfected with β-catenin siRNA or scrambled siRNA control; Caki-1 were transfected with WNT10A siRNA or scrambled siRNA control. In brief, 2 mL of 0.5% agarose in complete RPMI-1640 was used as the bottom agar in a 6-cm dish. A total of 2×10^4^ cells were mixed with 0.3% agarose in complete RPMI-1640 after 48 h of transfection. Cells were maintained in a humidified 5% CO_2_ incubator at 37°C for 15 days, with the old medium being replaced by a fresh medium every 3 days. For prolonging the ectopic effects of WNT10A and WNT10A siRNA, pcDNA-WNT10A-transfected 786-O were treated with 200 µg/mL G418 (Invitrogen); moreover, cells were continuously treated with 10 nM of indicated siRNA in a soft agar medium. On the 15th day, the cells were stained with crystal violet for 1 min and destained with tap water for 15 min. For each dish, colonies were counted using the Image J software. Each measurement was performed in triplicate.

### Data Analysis

Histoscores of BRD, paratumoral, and RCC tissues were subclassified as high (histoscores > mean of total samples of BRD, RCC, and paratumoral tissues) and low (histoscores ≤ mean of total samples of BRD, RCC, and paratumoral tissues) expression levels (mean values are listed in Figure 2B). Incidence risk was evaluated using logistic regression, and survival analysis was performed using Cox hazard regression model. Original real-time PCR data, western blot data, migration/invasion data, and colony formation data were recorded as continuous variables and analyzed using Student’s *t*-test or linear polynomial ANOVA with LSD post hoc examination. All the statistical analyses were performed using SPSS 16.0 and Excel 2007. All the statistical tests and *P* values were 2-sided, and the level of significance was set at <0.05 (*), <0.01 (**), or <0.001 (***).

## Results

### Expression Profiles of 19 *WNT* Genes from Kidney Cell Lines and Tissues

We comprehensively compared the individual expression profiles of 19 *WNT* genes from kidney cell lines and tissues by using RT-PCR (Table S1) [39]. Six cell lines were employed, i.e., 5 RCC cell lines 786-O, Caki-1, RCC-1, A498, and ACHN and 1 immortalized normal kidney proximal tubule epithelial cell line HK-2. Our results showed that WNT10A expression was significantly higher in most RCC cell lines (highly expressed in Caki-1, RCC-1, and ACHN and weakly expressed in 786-O and A498) but not in the HK-2 cell line. However, most other *WNT* genes were detected in both HK-2 and RCC cell lines (Figure 1A). RT-PCR comparison of 19 *WNT* genes was performed in 6 pairs of RCC tissues (T1–T6), normal kidney tissues in the proximity of RCC (paratumoral region, N1–N6), and 4 normal kidney tissues from BRD (N7–N10). The results of RT-PCR comparison showed that WNT10A expression was significantly higher in most RCC tissues than in normal kidney tissues (Figure 1B). These results were further confirmed by quantitative real-time PCR on an independent data set comprising 10 RCC and 10 normal kidney tissues (Figure 1C; primer sets for RT-PCR and quantitative real-time PCR are listed in Table S1). The results suggested that WNT10A may function as an oncogenic marker in RCC.

### WNT10A- and β-catenin-dependent Signaling is Associated with RCC Carcinogenesis and Poor Prognosis

WNT10A has been implicated to participate in canonical β-catenin-dependent signaling [40]. We evaluated the clinical significance of WNT10A- and β-catenin-dependent signaling molecules in a 19-year follow-up cohort comprising kidney tissues from 284 RCC and 267 BRD patients. Immunohistochemical profiles of WNT10A, β-catenin, and cyclin D1 were compared in normal kidney tissues from BRD, paratumoral tissues, and tumoral tissues from RCC. In BRD and paratumoral tissues, WNT10A showed a very weak cytoplasmic staining pattern in the epithelial cells of the renal tubules (Figure 2A, cortex and medulla). In RCC tissues, WNT10A showed a strong cytoplasmic and/or membranous staining pattern (Figure 2A, RCC). To confirm the WNT10A expression profile, another WNT10A antibody (sc-69135) and its competitive peptide were used for immunohistochemical analysis of serial sections. Similar results (Figure 2A, sc-69135) and related ablation profiles (Figure 2A, sc-69135P) were observed. WNT10A expression was significantly lower in most BRD and paratumoral tissues and significantly higher in most RCC tissues (Figure 2B (A, B); *P*_trend_ <0.001).

In BRD and paratumoral tissues, β-catenin showed a strong membranous staining pattern with minor cytoplasmic staining (Figure 2B(C)). However, β-catenin dramatically decreased in the cell membrane and increased both in the cytoplasm and nucleus of RCC cells (Figure 2B(D), red arrows indicate some representative nuclear-stained cells; *P*_trend_ <0.001). We also found that nuclear c-myc (Figure 2B(E,F)) and cyclin D1 expression was significantly increased in RCC tissues (Figure 2B(G,H)), *P*_trend_ <0.001 for both). These results suggest that WNT10A plays an oncogenic role in RCC in association with the β-catenin-dependent pathway.

Clinical significance of WNT10A, β-catenin, c-myc, and cyclin D1 in renal carcinogenesis was further evaluated using univariate and multivariate logistic regression. The results showed that WNT10A, nuclear β-catenin, and nuclear cyclin D1 were independent risk factors for renal carcinogenesis even after adjustment of age and gender and increased the incidence risk of renal carcinogenesis by 2–4 fold (WNT10A, 2.20 fold; nuclear β-catenin, 2.65 fold; and nuclear cyclin D1, 3.95 fold (Table 1)). However, cytoplasmic β-catenin and c-myc lost their significance after the adjustment of other risk factors (Table 1). As documented previously, men are at a higher risk of developing RCC than women in this recruited cohort [41]. Postoperative effects of these markers were further evaluated using survival assay in the RCC population. According to Table 2, WNT10A, nuclear β-catenin, and nuclear cyclin D1 also served as independent risk factors for higher RCC-associated death (WNT10A, 2.51-fold; nuclear β-catenin, 2.33-fold; and nuclear cyclin D1, 2.02-fold poor overall survival (OS); WNT10A, 2.73-fold; nuclear β-catenin, 2.42-fold; and nuclear cyclin D1, 2.40-fold poor disease free survival (DFS)). Cytoplasmic β-catenin and c-myc lost their significance after adjustment of other risk factors (Table 2). As reviewed previously [41], higher stage and higher grade of RCC were also associated with poor prognosis (Table 2). Higher WNT10A, nuclear β-catenin and nuclear cyclin D1 also revealed accumulated dose effects for higher RCC associated death (Figure 2C). These results indicate that WNT10A serves as an independent risk factor for RCC carcinogenesis and poor prognosis.

### WNT10A Activates β-catenin-dependent Signaling Pathway in Kidney and RCC Cells

WNT10A activation of β-catenin-dependent signaling pathway was further validated in a kidney cell line model. Forced expression of WNT10A was achieved in HK-2, 786-O, and A498 (low endogenous WNT10A), which significantly induced the proliferation of these cells. Conversely, we also examined RCC-1 and Caki-1 cells (high endogenous WNT10A) with loss-of-function design and observed that WNT10A siRNA knockdown in these cells significantly reduced their proliferation (Figure 3A). To clarify the specificity of WNT10A and β-catenin antibodies, the representative WNT10A and β-catenin gels with full molecular weight scales were also shown (Figure S2).

Intracellular translocation of β-catenin and upregulation of cyclin D1 and c-myc were observed in pcDNA-WNT10A-transfected HK-2, 786-O, and A498 (Figures 3A, 3B, and S3). Nuclear colocalization of β-catenin and cyclin D1 was also observed in pcDNA-WNT10A-transfected cells (Figure 3B). However, cotransfection of pcDNA-WNT10A and β-catenin siRNA exerted reverse effects on cyclin D1 and c-myc induction (Figure 3A). Conversely, WNT10A siRNA-transfected RCC-1 and Caki-1 showed decreased intracellular β-catenin, cyclin D1, and c-myc levels (Figure 3A, 3B). WNT10A-induced activation of β-catenin-dependent signaling was further confirmed using TCF/LEF reporter assay of 786-O and Caki-1. Forced WNT10A expression induced higher luciferase activity in 786-O than in controls; however, WNT10A siRNA knockdown reduced the luciferase activity in Caki-1 (Figure 3C). These results indicate that WNT10A promotes renal cell proliferation by activating WNT/β-catenin pathway.

### WNT10A Induces Higher Aggressiveness of RCC Cell Lines

Because β-catenin signaling is involved in several pathways of cancer aggressiveness [6], [9]–[11], we also performed some functional assays in RCC cell line model to investigate the oncogenic role of WNT10A. First, we analyzed the effect of WNT10A on RCC cell survival; however, both forced WNT10A expression and WNT10A siRNA knockdown had minor effects on cell cycle profiles, with only slightly induced G1 phase increment. As documented previously, β-catenin siRNA-transfected cells showed more obvious G1 arrest [42] than WNT10A siRNA-transfected cells. It was suggested that abolition of endogenous β-catenin expression caused a stricter G1 arrest than that caused by WNT10A siRNA-induced partial blockage of β-catenin signaling (Figure 4). We took advantage of β-catenin-induced chemoresistance [40] to determine the apoptotic effect of WNT10A. Forced WNT10A expression reduced the apoptotic effects of epirubicin and cisplatin in 786-O; however, each WNT10A siRNA treatment increased the sensitivity of these drugs in Caki-1 (Figure 4). β-Catenin siRNA knockdown increased the chemosensitivity of epirubicin and cisplatin toward both the cell lines (Figure 4). Cleaved caspase 3 and PARP were used as apoptotic markers to evaluate the chemosensitivity of pcDNA-WNT10A or only vector transfected 786-O and WNT10A siRNA or scrambled siRNA transfected Caki-1 cells. Forced WNT10A expression reduced epirubicin- and cisplatin-induced cleavage of caspase 3 and PARP; however, β-catenin siRNA cotransfection partially recovered the sensitivity of these compounds and increased epirubicin- and cisplatin-induced cleavage of caspase 3 and PARP. Conversely, both WNT10A siRNA and β-catenin siRNA increased the chemosensitivity of Caki-1; however, β-catenin siRNA induced higher cleavage of caspase 3 and PARP than WNT10A siRNA (Figure 5).

Second, we analyzed migration abilities by using wound-healing and transwell assays. Forced WNT10A expression increased the cell migration ability of 786-O and A498 (Figures 6, Figure 7); however, WNT10A siRNA knockdown reduced the migration ability of Caki-1 (Figures 6, Figure 7). Similar effects were observed for invasion assay with Matrigel-coated transwells (Figure 7). Third, cell transformion ability was evaluated using soft agar colony formation assay. The results showed that forced WNT10A expression increased the colony numbers of 786-O, and β-catenin siRNA transfected 786-O reduced the colony formation ability. Conversely, WNT10A siRNA knockdown reduced the colony numbers of Caki-1 (Figure 8). These results suggest that WNT10A promotes RCC aggressiveness by activating β-catenin-dependent pathway.

## Discussion

Previous studies have shown that promoters of most *WNT* antagonistic genes (*sFRP1* [28]–[30], *sFRP2* [31], *sFRP3* [32], *sFRP5* [33], *DKKs* [34], [35], and *WIF1* [36]) are methylated or epigenetically silenced in RCC, which results in uninhibited WNT signaling during cancer initiation or progression [26]. To the best of our knowledge, this is the first study to address the role of WNT ligands in RCC carcinogenesis and progression to date. We comprehensively screened mRNA expression profiles of 19 *WNT* genes and found that *WNT10A* was overexpressed in RCC cell lines and tissues but limitedly expressed in normal kidney controls.

*WNT10A* overexpression has been observed in some cancer cell lines, but its role in RCC is still unknown [34], [43], [44]. After binding to its receptors, WNT ligands prompt intracellular β-catenin accumulation and nuclear translocation leading to the activation of TCF/LEF signaling pathway, in addition to c-myc and cyclin D1 induction. Immunohistochemical profiles of the recruited cohorts showed that WNT10A, β-catenin, and cyclin D1 were highly expressed in RCC tissues. Higher β-catenin expression observed in our study was similar to that observed in previous studies [22]–[25]; this increased β-catenin expression in turn induced higher cyclin D1 expression in RCC cell nucleus. Retrospective comparison of 284 RCC and 267 BRD patients showed that WNT10A, nuclear β-catenin, and nuclear cyclin D1 serve as independent risk factors for RCC carcinogenesis and prognosis. Furthermore, in gain-of-function design, both normal kidney cell line (HK-2) and RCC cell lines (786-O and A498) showed increased intracellular β-catenin accumulation with cyclin D1 and c-myc upregulation due to forced WNT10A expression. Forced WNT10A expression in HK-2, 786-O, and A498 also resulted in increased cell proliferation, chemoresistance, migration, invasiveness, and cell transformation. Conversely, in the loss-of-function design, WNT10A siRNA knockdown in RCC-1 and Caki-1 decreased intracellular β-catenin accumulation, downregulated cyclin D1 and c-myc expression, and thus reduced cell proliferation, chemoresistance, migration, invasiveness, and cell transformation. These results indicate that WNT10A plays an oncogenic role in RCC carcinogenesis and aggressiveness by activating β-catenin-dependent pathway. Interestingly, there is no significant difference in cell cycle phase discerned between RCC cells with forced WNT10A expression and those with WNT10A siRNA knockdown; however, β-catenin siRNA-transfected cells showed more obvious G1 arrest [42] than RCC cells with elevated WNT10A or suppressed WNT10A. This result indicates that abolition of endogenous β-catenin expression caused a stricter G1 arrest than that caused by WNT10A siRNA-induced partial blockage of β-catenin signaling. Moreover, forced WNT10A expression had a profound effect on cell proliferation that was noticeable 72 h after ectopic WNT10A transfection. However, because flow cytometry was performed with higher density of cells (>70% confluence) over a shorter span (48 h after ectopic WNT10A transfection), which were resulted in slower cell proliferation rate and had lower accumulative effect of forced WNT10A expression. This might explain the unapparent difference in cell cycle phases of flow cytometry between cells with forced WNT10A expression and those transfected with vector controls.

WNT/β-catenin signaling is known to play a crucial role in carcinogenesis and progression of several types of cancers [8]; moreover, this signaling is also involved in stem cell regulation of skin cancer [45], intestinal cancer [46], and breast cancer [47]–[50]. In addition to the well-studied canonical WNT1, dysregulation of several WNT ligands induces aberrant WNT/β-catenin signaling in different cancers or tumors, with inconsistency of typical classification [7]. Different WNT/β-catenin signaling pathways are dysregulated in different types of tumors (Table S2). Previous studies have shown that different WNT ligands might play important roles in different cancers, mostly by activating β-catenin-dependent pathways. Besides, non-canonical WNT pathways, especially the typical WNT5A and WNT11 pathways, are reported to be involved in several carcinogenic and cancer progression pathways [12], [13], [51]. However, some WNT ligands are known to play inverse roles that are independent of β-catenin/TCF activation [52].

Although WNT ligands may be supplied by different cells present in the tumor microenvironment, autocrine WNT signaling has been reported to be dominant in several cancers without mutations in the WNT pathway-related genes, which results in cell proliferation or survival [53]. In this study, we found that WNT10A showed a strong immunohistochemical staining pattern in the cytoplasm of RCC cells; however, weak staining patterns were observed in cells from normal kidney tubes and stromal tissues. *WNT10A-WNT6* are known to cluster in a head-to-tail manner in the q35 region of chromosome 2 [44]. We did not observe any significant difference in *WNT6* mRNA expression between kidney and RCC cells or tissues. Combined with the results obtained from gain- and loss-of-function designs of cell line models, our results suggest that WNT10A might exert an autocrine effect in RCC, resulting in the induction of WNT/β-catenin signaling and promotion of cell proliferation.

In conclusion, the present study showed that WNT10A plays an oncogenic role in RCC through the canonical WNT/β-catenin signaling pathway, eventually promoting RCC carcinogenesis and aggressiveness. We found that WNT10A, nuclear β-catenin, and nuclear cyclin D1 serve as independent risk factors for RCC carcinogenesis and progression in the recruited RCC and BRD patients. To date, WNT ligands have not been evaluated in RCC. This study comprehensively screened *WNT* genes in RCC patients and cells and found that WNT10A plays carcinogenic and prognostic roles in RCC by activating β-catenin-dependent pathway. It also explained that RCC carcinogenesis and progression due to WNT/β-catenin signaling dysregulation is not only caused by β-catenin overactivation or WNT antagonist silencing but also caused by the autocrine effects of tumor-produced WNT ligands, especially WNT10A. Therefore, WNT10A may serve as an useful marker for RCC diagnosis and therapeutic improvement.

## Supporting Information

Figure S1 **Summary of cohort construction.** We recurited 3,494 individuals from TSGH Pathology Index or Cancer Registry Center. Any following criteria were excluded: (a) only pathological examinations or consults for other therapeutic institutes (n = 449) and other racial visitors (n = 6), (b) biopsy specimens which is too small for further examination, (c) refusal or confusion to allow use of their medical records for research, (d) only de-linked records available, (e) lost to follow-up after operation, (f) anyone of previous malignancy carrier were not involved to prevent unexpected influence, (g) non-RCC kidney cancers or other cancer metastate to kidney when re-diagnosis, (h) ambigious diagnosis in serial FFPE specimens, (i) specimens unusable or unavailable such as only stromal tissue rested in tissue microarray.
(PDF)

Figure S2 **The WNT10A and β-catenin gels with full molecular weight scales.** To clarify of the specificity of WNT10A and β-catenin antibodies, transfection of pcDNA-WNT10A, pcDNA3.1, or cotransfection with β-catenin siRNA were performed in HK-2 cell. Transfection of scrambled siRNA was also performed as negative control. Conversely, WNT10A siRNA or scambled siRNA were transfected in Caki-1 and RCC-1. Twenty micrograms of total protein extract from each cell line was loaded onto SDS-polyacrylamide gel and western blot analysis was performed. The full molecular weight scales were labeled as indicated, and the dominant bands of WNT10A (46 kDa), β-catenin (∼95 kDa) and α-tubulin (55 kDa) were showed. The pcDNA-WNT10A transfected HK-2 cell increased the WNT10A expression than vector control or β-catenin and scrambled siRNA co-transfected controls. Conversely, WNT10A siRNA transfected Caki-1 and RCC-1 obviously decreased the endogenous WNT10A than scrambled siRNA or reagent transfected controls.
(PDF)

Figure S3 **Immunocytochemistry of pcDNA-WNT10A transfected A498 cell.** After pcDNA-WNT10A transfected in A498, the expression of WNT10A, β-catenin, and cyclin D1 was observed by immunocytochemistry. WNT10A was significantly increased in transfected cells. β-catenin was highly intracellular accumulation of WNT10A transfected cells compared to the lower membranous expression in vector and reagent controls. Cyclin D1 also upregulated in the nucleus of pcDNA-WNT10A transfected cells compared to the lower cytoplasmic expression in vector and reagent controls.
(PDF)

Table S1 **WNT family gene primer set for RT-PCR and real-time PCR.**
(DOC)

Table S2 **Summary of WNT/β-catenin dysregulation in different tumor types.**
(DOC)

Table S3 **Summary of clinical data of RCC and BRD subjects.**
(DOC)
