# Supplementary figures and images for: WNT10A Plays an Oncogenic Role in Renal Cell Carcinoma by Activating WNT/β-catenin Pathway
Source: PLoS One. 2012 Oct 19;7(10):e47649. doi: 10.1371/journal.pone.0047649 (PMC3477117; doi:10.1371/journal.pone.0047649)

Figure S1

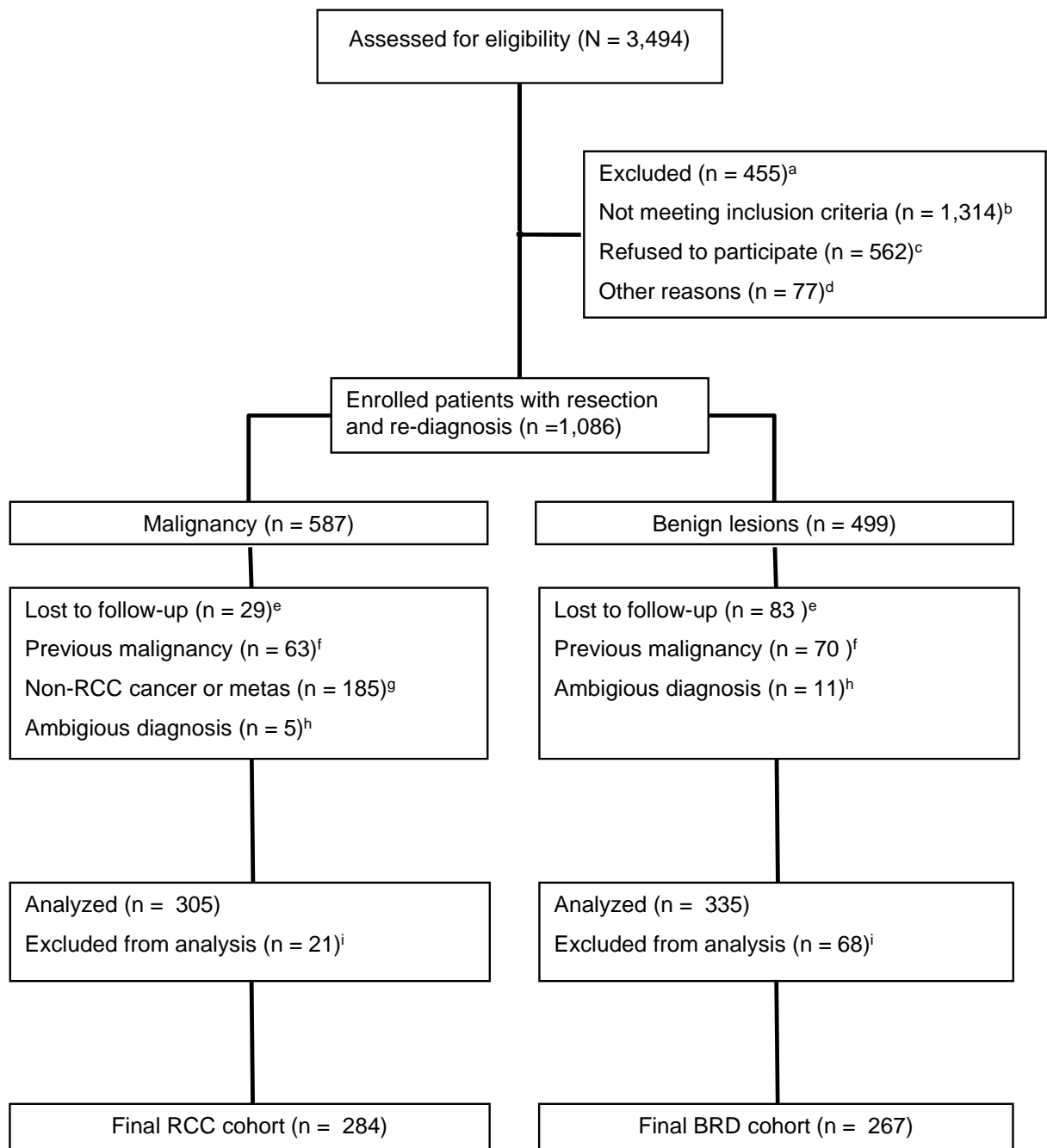

Supplement: Figure S1 — Summary of cohort construction. We recurited 3,494 individuals from TSGH Pathology Index or Cancer Registry Center. Any following criteria were excluded: (a) only pathological examinations or consults for other therapeutic institutes (n = 449) and other racial visitors (n = 6), (b) biopsy specimens which is too small for further examination, (c) refusal or confusion to allow use of their medical records for research, (d) only de-linked records available, (e) lost to follow-up after operation, (f) anyone of previous malignancy carrier were not involved to prevent unexpected influence, (g) non-RCC kidney cancers or other cancer metastate to kidney when re-diagnosis, (h) ambigious diagnosis in serial FFPE specimens, (i) specimens unusable or unavailable such as only stromal tissue rested in tissue microarray. (PDF) [file pone.0047649.s001.pdf]

Figure S2

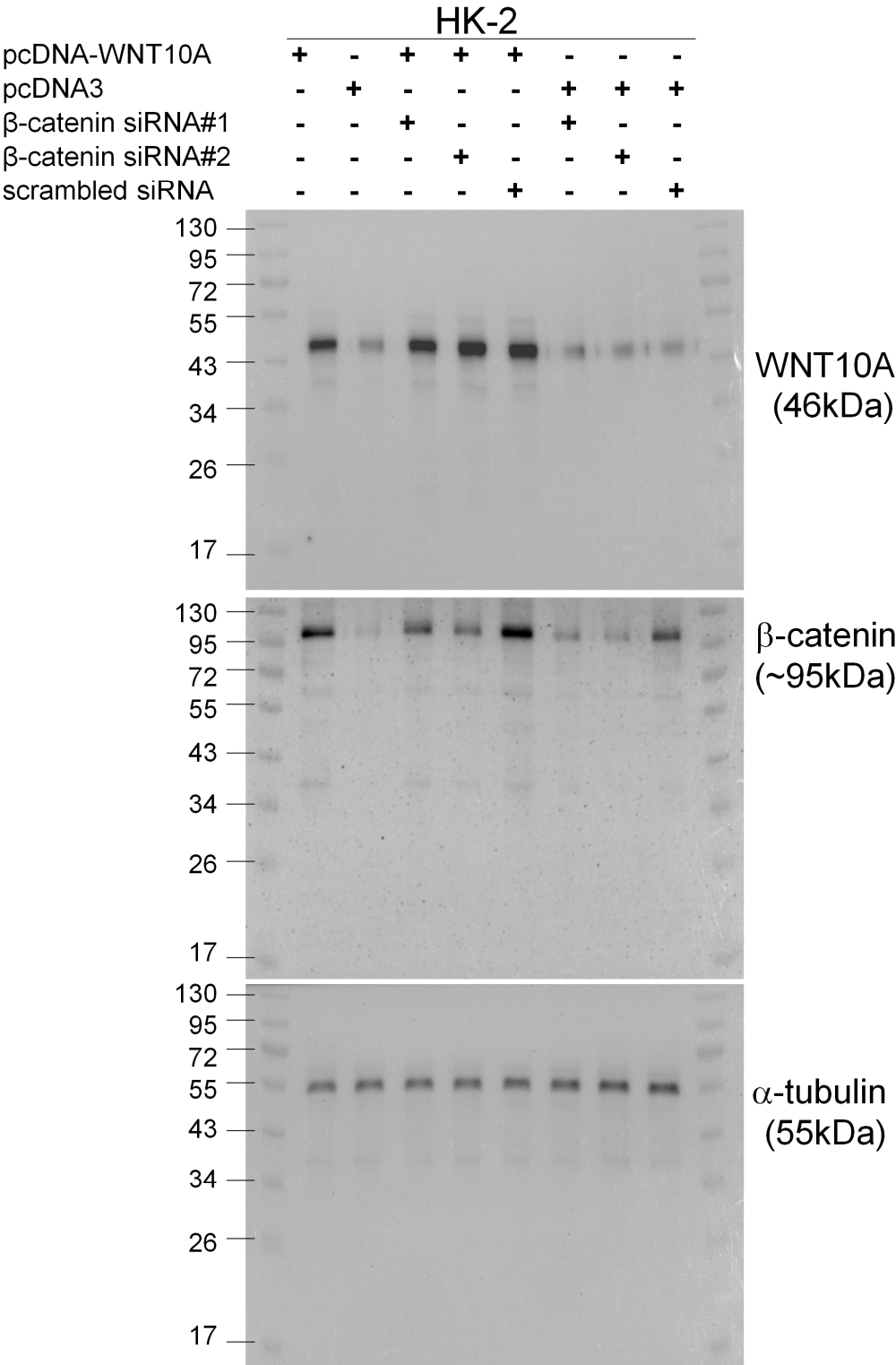

|                 | Caki-1 |   |   |   | RCC-1 |   |   |   |
|-----------------|--------|---|---|---|-------|---|---|---|
| Reagent control | +      | - | - | - | +     | - | - | - |
| scrambled siRNA | -      | + | - | - | -     | + | - | - |
| WNT10A siRNA#1  | -      | - | + | - | -     | - | + | - |
| WNT10A siRNA#2  | -      | - | - | + | -     | - | - | + |

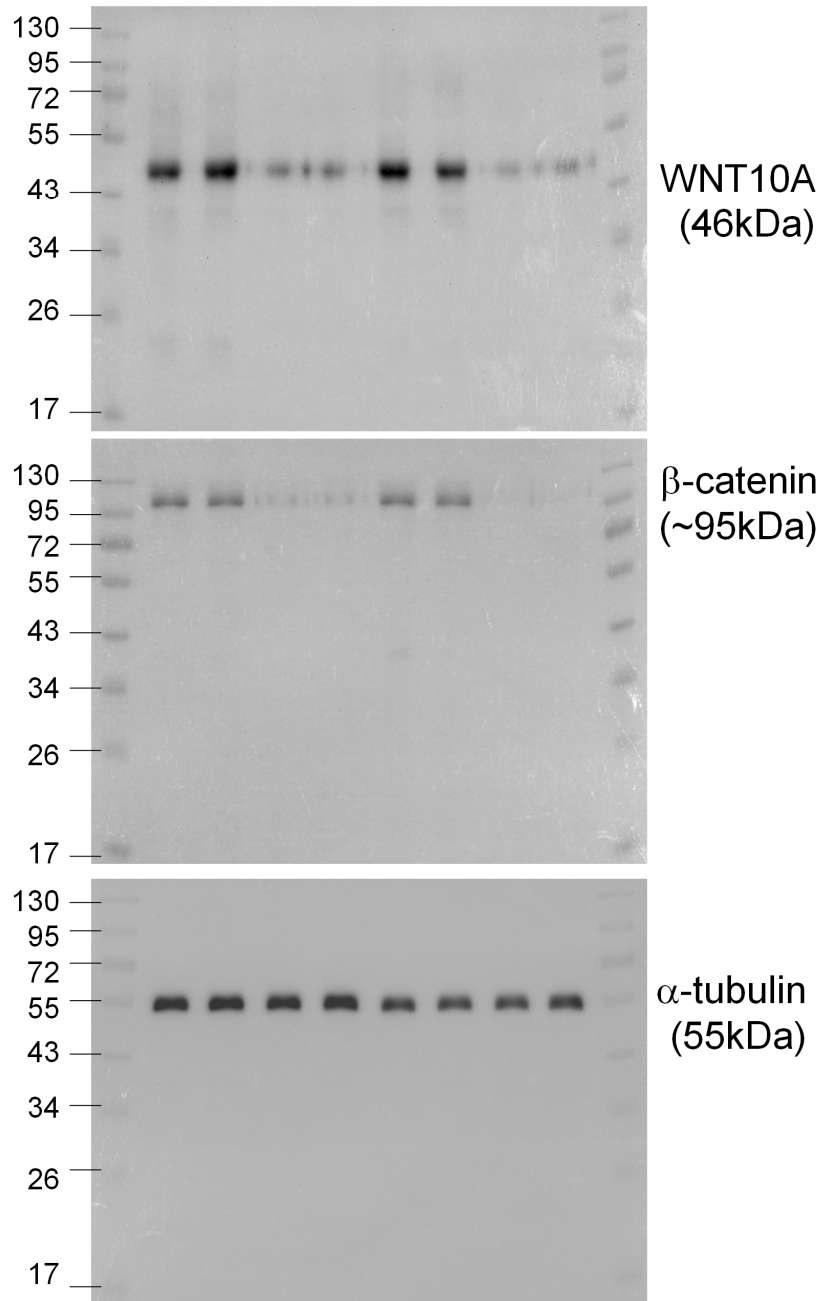

Supplement: Figure S2 — The WNT10A and β-catenin gels with full molecular weight scales. To clarify of the specificity of WNT10A and β-catenin antibodies, transfection of pcDNA-WNT10A, pcDNA3.1, or cotransfection with β-catenin siRNA were performed in HK-2 cell. Transfection of scrambled siRNA was also performed as negative control. Conversely, WNT10A siRNA or scambled siRNA were transfected in Caki-1 and RCC-1. Twenty micrograms of total protein extract from each cell line was loaded onto SDS-polyacrylamide gel and western blot analysis was performed. The full molecular weight scales were labeled as indicated, and the dominant bands of WNT10A (46 kDa), β-catenin (∼95 kDa) and α-tubulin (55 kDa) were showed. The pcDNA-WNT10A transfected HK-2 cell increased the WNT10A expression than vector control or β-catenin and scrambled siRNA co-transfected controls. Conversely, WNT10A siRNA transfected Caki-1 and RCC-1 obviously decreased the endogenous WNT10A than scrambled siRNA or reagent transfected controls. (PDF) [file pone.0047649.s002.pdf]

Figure S3

**A498**

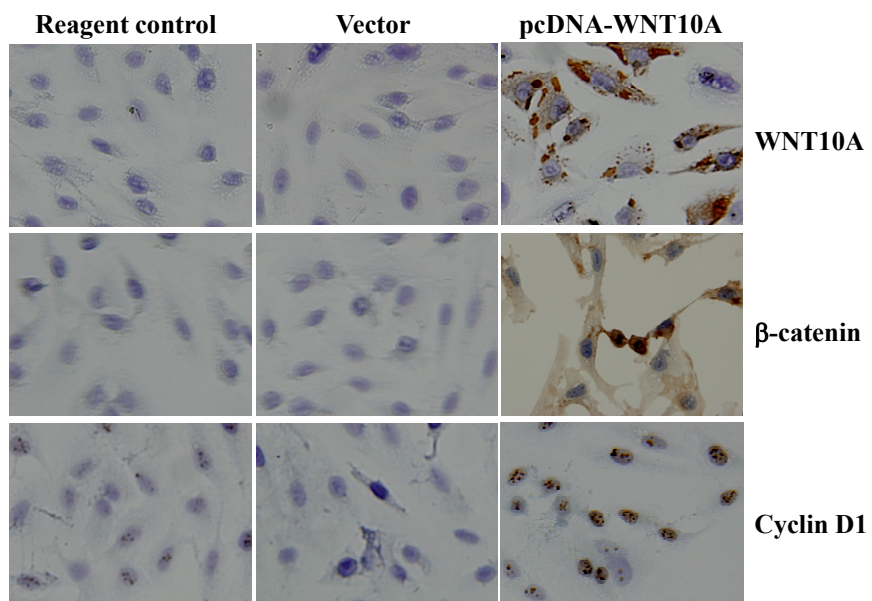

Supplement: Figure S3 — Immunocytochemistry of pcDNA-WNT10A transfected A498 cell. After pcDNA-WNT10A transfected in A498, the expression of WNT10A, β-catenin, and cyclin D1 was observed by immunocytochemistry. WNT10A was significantly increased in transfected cells. β-catenin was highly intracellular accumulation of WNT10A transfected cells compared to the lower membranous expression in vector and reagent controls. Cyclin D1 also upregulated in the nucleus of pcDNA-WNT10A transfected cells compared to the lower cytoplasmic expression in vector and reagent controls. (PDF) [file pone.0047649.s003.pdf]
