# Supplementary material for: WNT10A Plays an Oncogenic Role in Renal Cell Carcinoma by Activating WNT/β-catenin Pathway
Source: PLoS One. 2012 Oct 19;7(10):e47649. doi: 10.1371/journal.pone.0047649 (PMC3477117; doi:10.1371/journal.pone.0047649)
Supplement: Table S1 — WNT family gene primer set for RT-PCR and real-time PCR. (DOC) [file pone.0047649.s004.doc]

| **Table S1. WNT family gene primer set for RT-PCR** | | | |
| --- | --- | --- | --- |
| **Gene** |  | **Sequence (5` to 3`) [39]** | **Amplicon (bp)** |
| WNT1 | F | gcgtctgatacgccaaaatc | 244 |
|  | R | ggattcgatggaaccttctg |  |
| WNT2 | F | tagtcgggaatctgcctttg | 221 |
|  | R | ttcctttcctttgcatccac |  |
| WNT2B | F | ctcatcagcaggggtagtcc | 159 |
|  | R | aaaacggacaccgtagtgga |  |
| WNT3 | F | acgagaactcccccaacttt | 170 |
|  | R | gatgcagtggcatttttcct |  |
| WNT3A | F | tgttgggccacagtattcct | 302 |
|  | R | atgagcgtgtcactgcaaag |  |
| WNT4 | F | ccttcgtgtacgccatctct | 250 |
|  | R | gcctcattgttgtggaggtt |  |
| WNT5A | F | ccacatgcagtacatcggag | 378 |
|  | R | cactctcgtaggagcccttg |  |
| WNT5B | F | gtgcagagacccgagatgtt | 550 |
|  | R | caggctacgtctgccatctt |  |
| WNT6 | F | ggttatggaccctaccagca | 208 |
|  | R | aatgtcctgttgcaggatgc |  |
| WNT7A | F | agtacaacgaggccgttcac | 326 |
|  | R | gcacgtgttgcacttgacat |  |
| WNT7B | F | aagctcggagcactgtcatc | 374 |
|  | R | ccctcggcttggttgtagta |  |
| WNT8A | F | tggggaacctgtttatgctc | 456 |
|  | R | ccctcggcttggttgtagta |  |
| WNT8B | F | ctggtccaaaggcttacctg | 557 |
|  | R | tgagtgctgcgtggacttc |  |
| WNT9A | F | gacggtcaagcaaggatctg | 411 |
|  | R | tgctctcgcagttcttctca |  |
| WNT9B | F | ctgcttgagtgccagtttca | 477 |
|  | R | cgagtcatagcgcagtttca |  |
| WNT10A | F | aatgccaacaccaattcagg | 464 |
|  | R | caactcggttgttgtgaagc |  |
| WNT10B | F | gcaagagtttcccccactct | 367 |
|  | R | gattgcggttgtgggtatc |  |
| WNT11 | F | ttgcttgacctggagagagg | 521 |
|  | R | gacgagttccgagtccttca |  |
| WNT16 | F | tgctccgatgatgtccagta | 562 |
|  | R | acctcctgcaacggacatag |  |

| **WNT family gene primer set for real time PCR** | | | | |
| --- | --- | --- | --- | --- |
| **Gene** |  | **Sequence (5` to 3`)** | **Amplicon (bp)** | **Reference** |
| WNT1 | F | ctcatgaaccttcacaacaacga | 80 | [54] |
|  | R | atcccgtggcacttgca |  |  |
| WNT2 | F | cctgatgaatcttcacaacaacaga | 78 | [54] |
|  | R | ccgtggcacttgcactctt |  |  |
| WNT2B | F | tgccaaggagaagaggcttaag | 68 | [55] |
|  | R | gtgcgaccacagcggttatt |  |  |
| WNT3 | F | tgtgaggtgaagacctgctg | 207 | [56] |
|  | R | aaagttgggggagttctcgt |  |  |
| WNT3A | F | gccccactcggatacttcttact | 98 | [54] |
|  | R | gaggaatactgtggcccaaca |  |  |
| WNT4 | F | catgcaacaagacgtccaag | 121 | [56] |
|  | R | aagcagcaccagtggaattt |  |  |
| WNT5A | F | gggaggttggcttgaacata | 141 | [56] |
|  | R | gaatggcacgcaattacctt |  |  |
| WNT5B | F | ctgcctttccagcgagaatt | 77 | [55] |
|  | R | aggtcaaatggcccccttt |  |  |
| WNT6 | F | tccgccgctggaattg | 74 | [57] |
|  | R | aggccgtctcccgaatgt |  |  |
| WNT7A | F | ggagggtccttttcctgggt | 190 | [56] |
|  | R | atattgctgtgatgaggccc |  |  |
| WNT7B | F | gcaagtggattttctacgtgtttct | 65 | [54] |
|  | R | tgacagtgctccgagcttca |  |  |
| WNT8A | F | gcagaggcggaactgatctt | 85 | [57] |
|  | R | cgaccctctgtgccatagatg |  |  |
| WNT8B | F | ttcccaagaatcttgaatgc | 142 | [56] |
|  | R | actccagagctccctcttcc |  |  |
| WNT9A | F | cttaagtacagcagcaagttcgtcaa | 93 | [55] |
|  | R | ccacgaggttgttgtggaagt |  |  |
| WNT9B | F | caggtgctgaaactgcgctat | 65 | [55] |
|  | R | gcccaaggcctcattggt |  |  |
| WNT10A | F | ggcaacccgtcagtctgtct | 62 | [55] |
|  | R | cattccccacctcccatct |  |  |
| WNT10B | F | gcgccaggtggtaactgaa | 59 | [54] |
|  | R | tgcctgatgtgccatgaca |  |  |
| WNT11 | F | ggcttgtgctttgccttca | 77 | [55] |
|  | R | tttgatgtcctgccctcctt |  |  |
| WNT16 | F | cgggagccagttcagacacga | 238 | [58] |
|  | R | cacttgctgagccgccgttct |  |  |
