# Supplementary material for: WNT10A Plays an Oncogenic Role in Renal Cell Carcinoma by Activating WNT/β-catenin Pathway
Source: PLoS One. 2012 Oct 19;7(10):e47649. doi: 10.1371/journal.pone.0047649 (PMC3477117; doi:10.1371/journal.pone.0047649)
Supplement: Table S2 — Summary of WNT/β-catenin dysregulation in different tumor types. (DOC) [file pone.0047649.s005.doc]

| **Table S2 Summary of WNT/β-catenin dysregulation in different tumor types** | |
| --- | --- |
| WNT member | Tumor types |
| WNT1 | Lung cancer, breast cancer, prostate cancer, glioma, and several cancer types (reviewed in [59]) |
| WNT2 | Lung cancer [60], melanoma [61], gastric cancer [62-63], esophageal cancer [64-65], glioma [66], and colorectal cancer [67] |
| WNT2B (WNT13) | Gastric cancer [68-69] |
| WNT3 | Leukemia or lymphoma [15,57,70], squamous cell carcinoma [71], liver cancer [72-74], and breast cancer [75-76] |
| WNT3A | Prostate cancer [77], leukemia [70, 77-79] and melanoma [80] |
| WNT4 | Pituitary adenomas [81] |
| WNT5B | Leukemia [57] |
| WNT6 | Leukemia [57] and neuroblastoma [82] |
| WNT7A | Neuroblastoma [82], endometrial cancer [83], and ovarian cancer [84] |
| WNT7B | Mammary tumor [75] and prostate cancer [85] |
| WNT9A (WNT14) | Leukemia [57] |
| WNT10A | Leukemia or lymphoma [57,15] |
| WNT10B | Neuroblastoma [82], osteosarcoma [86], liver cancer [87], and breast cancer [88-89] |
| WNT16 | Leukemia or lymphoma [68, 90-91] |
