# Supplementary material for: WNT10A Plays an Oncogenic Role in Renal Cell Carcinoma by Activating WNT/β-catenin Pathway
Source: PLoS One. 2012 Oct 19;7(10):e47649. doi: 10.1371/journal.pone.0047649 (PMC3477117; doi:10.1371/journal.pone.0047649)
Supplement: Table S3 — Summary of clinical data of RCC and BRD subjects. (DOC) [file pone.0047649.s006.doc]

Table S3 Summary of clinical data of RCC and BRD subjects

|  | **RCC (n = 284)** | **BRD (n = 267)** |
| --- | --- | --- |
| Age (mean±S.D.) | 59.44±13.79 | 53.86±18.52 |
| Sex |  |  |
| Female | 98 (34.5%) | 141 (52.8%) |
| Male | 186 (65.5%) | 126 (47.2%) |
| Histologic grade |  | NA |
| G1 | 14 (4.9%) |  |
| G2 | 103 (36.3%) |  |
| G3 | 108 (38.0%) |  |
| G4 | 59 (20.8%) |  |
| Tumor size |  | NA |
| T1 | 119 (41.9%) |  |
| T2 | 64 (22.5%) |  |
| T3 | 86 (30.3%) |  |
| T4 | 15 (5.3%) |  |
| Nodal invasion |  | NA |
| N0 | 262 (92.3%) |  |
| N1 | 12 (4.2%) |  |
| N2 | 10 (3.5%) |  |
| Metastasis |  | NA |
| M0 | 255 (89.8%) |  |
| M1 | 29 (10.2%) |  |
| Stage |  | NA |
| Stage I | 108 (38.0%) |  |
| Stage II | 55 (19.4%) |  |
| Stage III | 75 (26.4%) |  |
| Stage IV | 46 (16.2%) |  |
| Histology |  | NA |
| CCRCC | 230 (81.0%) |  |
| PRCC | 34 (12.0%) |  |
| ChRCC | 20 (7%) |  |
| Lateriality |  |  |
| Left | 158 (55.6%) |  |
| Right | 126 (44.4%) |  |
| Chemicotherapy |  | NA |
| + | 26 (9.2%) |  |
| - | 258 (90.8%) |  |
| Radiotherapy |  | NA |
| + | 52 (18.3%) |  |
| - | 232 (81.7%) |  |
| Survival |  | NA |
| Survival | 202 (71.1%) |  |
| Expired | 82 (28.9%) |  |
